# Follicular Helper T Cells and B Cell Maturation in Patients with 22q11.2 Deletion Syndrome and Recurrent Infections

**DOI:** 10.1007/s10875-026-01987-2

**Published:** 2026-02-03

**Authors:** Nouf Alsaati, Katherine Beigel, Kelly Maurer, Sarah E. Henrickson, Montana Knight, Audrey Green, Victoria Giunta, Daniel E. McGinn, Bekah Wang, T. Blaine Crowley, Donna M. McDonald-McGinn, Kathleen E. Sullivan

**Affiliations:** 1 Division of Allergy and Immunology, Children’s Hospital of Philadelphia, Philadelphia, PA 19104 USA; 2 Department of Biomedical and Health Informatics, Children’s Hospital of Philadelphia, Philadelphia, PA 19104 USA; 3 Division of Human Genetics, Children’s Hospital of Philadelphia, Philadelphia, PA 19104 USA; 4 Division of Allergy Immunology, ARC 1216-I, 3615 Civic Center Blvd., Philadelphia, PA 19104 USA

**Keywords:** 22q11.2 deletion syndrome, DiGeorge syndrome, T cell senescence, Follicular helper t cell, Atypical b cells

## Abstract

**Purpose:**

22q11.2 Deletion Syndrome has been primarily described as a disorder of T cell production secondary to thymic hypoplasia. However, there is great complexity in the clinical picture with infections, autoimmunity, and inflammation occurring. Emerging evidence suggests that qualitative T cell dysfunction occurs, and the goal of this study was to utilize single-cell RNA-seq to better define altered gene expression patterns to inform on the mechanisms associated with recurrent infections.

**Methods:**

We utilized single-cell RNA-seq to define distinct populations in 22q11.2 Deletion Syndrome (*N* = 13) and controls (*N* = 11) as well as within a subcohort of patients with 22q11.2 Deletion Syndrome and recurrent infections.

**Results:**

When we analyzed differentially expressed genes, we identified a signature of type I interferons across all cell types. Within the T cell compartment, and particularly within the follicular helper T cells, we identified a senescence signature. The alterations found in T cells were most substantial in the patients with recurrent infection.

**Conclusions:**

While T cell numbers can often normalize in patients with 22q11.2 Deletion Syndrome, our data indicate significantly altered function as defined by differentially expressed genes and aligned with what is known about T cell senescence. The effect was greatest in the patients with recurrent infection. This would be expected to impact T cell function and may account for ongoing symptoms, reduced B cell maturation, and possibly the risk of immune dysregulation.

**Supplementary Information:**

The online version contains supplementary material available at 10.1007/s10875-026-01987-2.

## Introduction

22q11.2 Deletion Syndrome (22q11.2DS) is one of the most common copy number variants in the general population with a birth prevalence of 1:2148 [7, 26, 41]. Immunodeficiency is common and T cell lymphopenia is the hallmark of 22q11.2DS due to thymic hypoplasia [1, 18]. Thymic aplasia occurs infrequently and the thymic phenotype can be conceptualized as a spectrum ranging from normal size to full aplasia with the majority of infants having some degree of thymic hypoplasia and therefore diminished T cell numbers [3, 28, 29, 43]. Most studies have focused on the enumeration of T cells as strategy to characterize the level of immunocompromise and, in infancy, this strategy can define patients requiring a thymic implant, those with essentially normal T cell numbers, and infants with mild to severe T cell compromise, with each of those strata defining some management choices [2, 9, 15, 53]. T cell counts are usually normal in adulthood, yet infections and risk of autoimmunity persist, suggesting T cell dysfunction is not adequately captured by T cell counts later in life [20, 46, 50, 57].

In 22q11.2DS, adulthood is accompanied by qualitative changes in the B cells and the appearance of antibody dysfunction in a subset of patients [24, 46, 47, 55]. The antibody dysfunction may manifest as specific antibody deficiency, hypogammaglobulinemia, or common variable immunodeficiency (CVID) with immune dysregulation [6, 24, 62, 65]. The antibody dysfunction is a treatable facet of 22q11.2DS but there are few biomarkers and even less mechanistic understanding of which patients have this less common but treatable evolution.

This clinical B cell dysfunction in 22q11.2DS has been recognized since the early 2000s and multiple studies have identified a subset of patients with either antibody dysfunction and/or altered maturation of B cells, characterized by low numbers of class-switched B cells (CSM) [14, 21, 46, 47, 74]. Low CSM B cells have been identified as a biomarker for immune dysregulation and B cell lymphopenia has also been linked to autoimmunity, supporting these findings as medically important [15, 49]. There have been fewer studies linking evolving B cell dysfunction with infections although this concept aligns with our understanding of CVID, with which these patients share some features, including the reduction in CSM B cells [72]. We undertook this study of B cells and T cells in 22q11.2DS to better understand the qualitative features associated with recurrent infections. We utilized single-cell RNA-sequencing (scRNA-seq) to deeply phenotype cells from patients and controls. We demonstrate a highly altered follicular helper T cell (Tfh) population with increased numbers of cells and exhibiting evidence of senescence.

## Methods

### Patients and Controls

This study was approved by the Institutional Review Board at Children’s Hospital of Philadelphia and patients and controls consented for participation. Thirteen subjects 8 years of age and older with 22q11.2DS, followed at Children’s Hospital of Philadelphia represented the 22q11.2DS study cohort. Patients were recruited as semi-consecutive patients (based on clinic availability and age requirements). Eleven healthy controls were age and sex matched (Table 1) as a group to the patients. The healthy donors were collected as explicitly completely healthy donors and patients self declared as healthy on the day of the blood draw. All samples were frozen and thawed according to the 10X Genomics protocol. The 22q11.2DS study cohort was evenly divided between patients with recurrent infections (*N* = 6) and those without (*N* = 7). Both cohorts had a median age of 11 years. We defined recurrent infections through record review and multiple visits listing infections. Supplemental Table 1 displays the relevant clinical and laboratory features of the patients.

**Table 1:** Study cohort characteristics

Characteristic	22q11.2 DS N = 3	Control N = 11
Sex		
Female	6 (46%)	5 (45%)
Male	7 (54%)	6 (55%)
Race		
White	8 (62%)	5 (45%)
Black	4 (31%)	1 (91%)
Other	1 (7-7%)	2 (18%)
Unknown	0 (0%)	3 (27%)
Age	15 (8-41)	14 (6-25)
Age bracket		
5-10 years	6 (46%)	4 (36%)
11-16 years	5 (38%)	4 (36%)
17 years and older	2 (15%)	3 (27%)
Recurrent infections		
N/A	0 (0%)	11 (100%)
No recurrent infections	7 (54%)	0 (0%)
Recurrent infections	6 (46%)	0 (0%)
Autoimmunity	0 (0%)	0 (0%)
n (%); Mean (Min-Max)		

### Flow Cytometry

Sixteen subjects with 22q11.2DS, age range of 10–39 years (mean age of 16.7 years) with 16 age-matched healthy controls (age range 9–38 years with mean age of 19.7 years) represented the flow cytometry cohort. Tfh cells were defined flow cytometrically using physical parameters, CD3+, CD4+, CXCR5 + and ICOS+. The reported percentages are Tfh within the CD4 population.

### ScRNA-seq

Single cell suspensions of cells were prepared after PBMC isolation using Ficoll-Paque. ScRNA-seq was performed on 20,000 cells using the Chromium 3’ single cell library kit (10X Genomics, Pleasanton, CA). Cells were run on a 10X Genomics Chromium X instrument and libraries were run on the Illumina NovaSeq 6000.

### Alignment and Quantification

CellRanger v7.1.0 was utilized for alignment and quantification of reads. GRCh38-2020-A was used as the reference genome for alignment, with defaults set for intronic inclusion and Single Cell 3’v3.

### Pre-processing and Integration

In R (v4.4.0), Seurat (v5.1.0) was used for downstream analysis of all PBMC scRNA-sequencing patient (*N* = 13) and control (*N* = 11) samples. Filtered feature-barcode matrices (from CellRanger) of all patient and control samples were merged using the SeuratObject (v5.0.2) merge function. The merged Seurat object was filtered to keep cells with nCount_RNA > 800, nFeature_RNA > 500, and mitochondrial content < 10%, and the Seurat standard approach with defaults parameters was used to normalize data, identify highly variable features, and scale data. Next, principal component analysis (PC) was run on the scaled data of the Seurat object. The R package *harmony* (1.2.0) was used to integrate all data using the PCA as the reduction. After integration with *harmony*, the *harmony* reduction (first 30 dimensions) was used to calculate UMAP embeddings and find neighbors for the integrated data. Additional information on methods is available in the Supplemental Methods.

### Clustering

Clusters were identified at a range of resolutions (0, 0.05, and 1 to 1.3 at intervals of 0.1) using Seurat’s FindClusters. The results from the range of clustering resolutions were used in *clustree* (v0.5.1) to identify the optimal clustering resolution based on which resolution was the most stable (i.e., similar to adjacent resolutions). This clustering process was repeated on each subset of the data to better refine clusters and identify specific cell types.

### Cell Type Annotation

Annotation of clusters was performed by examining marker expression and differentially expressed genes (from results of Seurat’s FindAllMarkers, calculated for each cluster). First, major cell type groups of interest were identified (T cells and B cells). The pre-processing, integration, and clustering steps were repeated for each subset Seurat object. The cell type specific expression is shown as heat maps displaying the top 10 cell type specific markers (Supplemental data Fig. 1). Each cell type, as expected, has a unique gene expression pattern. Violin plots for B cells markers are shown in Supplemental Fig. 2 and for T cells in Supplemental Fig. 3.

**Fig. 1:**
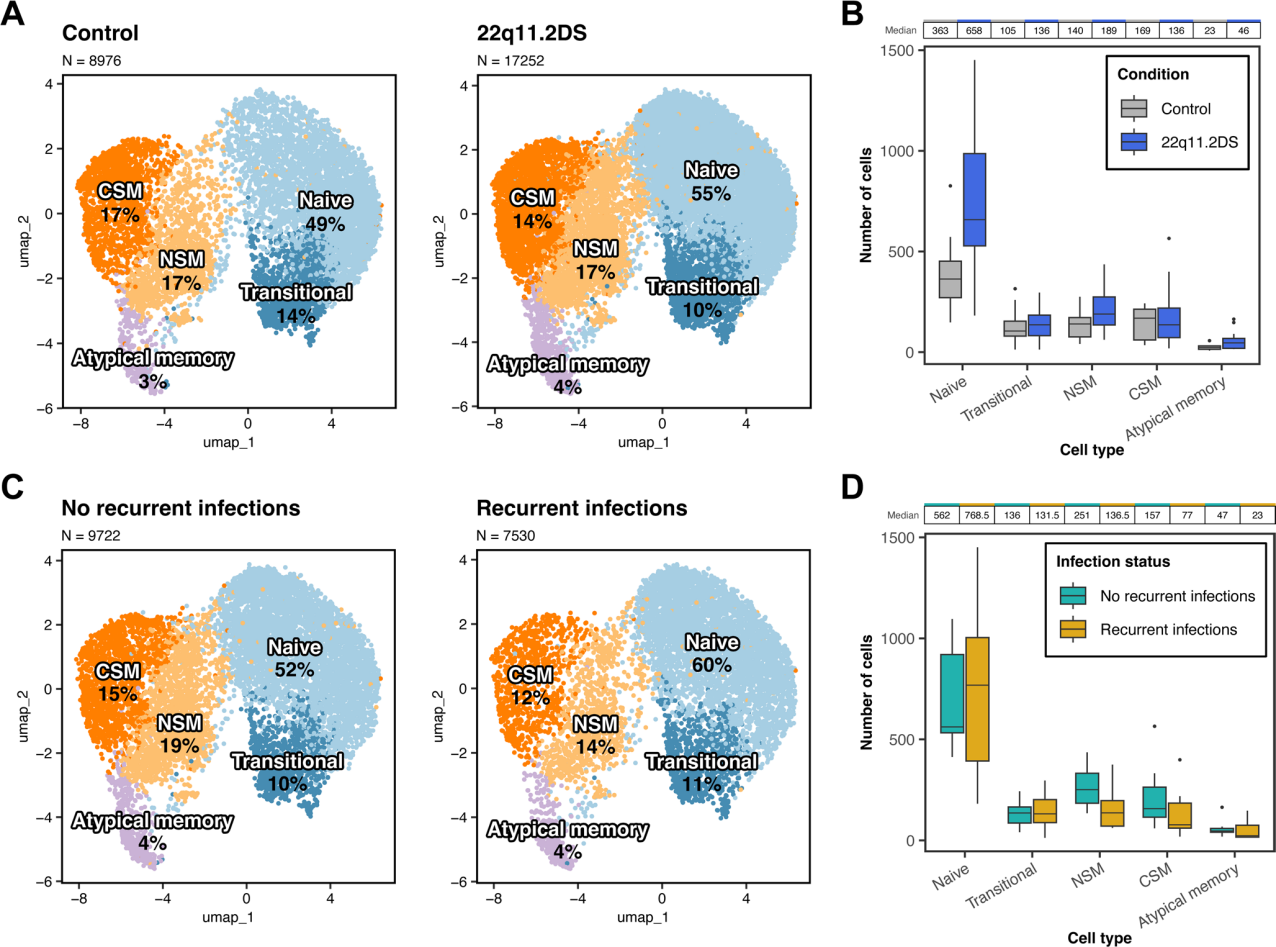
B cell analysis. 1**A**) B cell subsets were defined using the gene expression signatures indicated in Supplemental Table 2. The UMAP plots demonstrate the shift towards naïve memory B cells and away from CSM B cells in 22q11.2DS. Atypical memory B cells are higher in the patients with 22q11.2DS. This panel displays populations as percentages of the total B cell population. 1**B**) Box plots displaying B cell population numbers in the 22q11.2DS and control cohorts. Numbers of cells defined by scRNA-seq are reported on the y-axis. The horizontal line in the box and whisker plots represents the median for the population. The median is listed above the graph. 1**C**) B cell subsets differ in patients with 22q11.2DS depending on their history of recurrent infections. The subjects with recurrent infections had lower CSM B cells than those without. This panel displays populations as percentages of the total B cell population. 1**D**) Box plots comparing differentially distributed B cell subset numbers in patients with 22q11.2DS depending on their history of recurrent infections. Numbers of cells defined by scRNA-seq are reported on the y-axis. The horizontal line in the box and whisker plots represents the median for the population. The median is listed above the graph. CSM = class-switched memory B cells, NSM = non-switched memory B cells

### B Cells

The B cell clusters (from optimal resolution 1.0, based on the Clustree comparison of resolutions) were defined as B cells based on positive CD19 and MS4A1 expression indicated by violin and feature plots as well as average log2 fold change (FC) from FindAllMarkers. To define B cell subsets, the pre-processing, integration, and clustering steps were repeated. Naïve B cells, transitional B cells, non-switched memory (NSM, also called IgM memory), CSM B cells, and atypical memory B cells were identified based on marker expression as outlined in Supplemental Table 2 [63, 70].

### T Cells

The T cell clusters were identified from the total PMBC data set by expression of CD3E and CD3D. Clusters were defined as T cells based on violin and feature plots as well as positive CD3E and CD3D expression indicated by average log2FC from FindAllMarkers. To define T cell subsets, pre-processing, integration, and clustering steps were repeated for the T cell population. The T cells were then annotated as CD4+, CD8+ (based on expression plots and expression level in each cluster as indicated by average log2FC from FindAllMarkers) and double positive T cells. The double positive T cells represented a small population and was not further considered. Naïve, memory, and T regulatory clusters were identified. To further identify specific CD4 helper T cells, the CD4 memory T cells were subsetted and the pre-processing, integration, and clustering steps were repeated. Th1, Th2, Th17, and Tfh cells were identified based on marker expression as outlined in Supplemental Table 2 [16].

### Statistical Analyses

Differences between overall cell population distribution were calculated using Pearson’s Chi-squared test. Wilcoxon Rank Sum tests were used to test for differences in each cell type population between cohorts. Differences in gene expression were calculated using FindMarkers in Seurat, which uses the Wilcoxon Rank Sum test method and Bonferroni correction for p-values. P values of *≤* 0.05 were considered significant. Expression level differences for genes were calculated as log2FC and differences *≥* 0.25 were reported.

## Results

### Cohort Characteristics

Our patient cohort consisted of 13 patients with 22q11.2DS and the 11 controls were selected from a healthy donor pool to match for age and sex. Table 1 demonstrates that males and females were approximately equivalent in both groups and our racial distribution was comparable between patients and controls. Most of the subjects enrolled were school age and the mean of the patients’ ages was 15 years (median = 11 years) with the mean of the controls being 14 years (median = 12 years). Among the patients with 22q11.2DS, 6 had recurrent infections defined through record review as multiple visits with antibiotic use within one year (Supplemental Table 1). The mean age of the patients with recurrent infection was 11.7 years (median = 11 years) and the mean was 18.7 years (median = 11 years) for those with 22q11.DS without recurrent infections. No patients or controls had autoimmune disease. One patient was on immunoglobulin replacement for specific antibody deficiency.

### B Cell Analysis

We and others have previously identified lower levels of CSM B cells in older patients with 22q11.2DS compared to healthy donors [14, 15, 49]. We analyzed our scRNA-seq data to ensure our overall data aligned with current knowledge. The overall distribution of B cell populations defined by scRNA-seq was significantly different between patients and controls (Chi square *p* < 0.0001) with the altered distribution being reflected in higher percentages of naïve B cells, higher transitional B cells, higher NSM B cells and lower CSM B cells in patients with 22q11.2DS (percentages are given in Fig. 1A, cell counts defined by scRNA-seq are given in Fig. 1B). However, according to Wilcoxon Rank Sum tests, the only difference in an individual B cell population that reached statistical significance between patients and controls was the transitional B cell subset (Wilcoxon Rank Sum Test, *p* = 0.0474). The p value for naïve B cells was 0.15, for non-switched memory B cells was 0.169, and atypical memory cells was 0.303 (all by Wilcoxon analysis). The atypical memory B cells are those which are extensively antigen exposed and are often defined using flow cytometry as CD21lo [48, 72]. To define any possible age effects, we analyzed the data using the age brackets in Table 1 and found no difference according to age.

We then compared the patients with 22q11.2DS and recurrent infections to patients with 22q11.2DS without recurrent infections to identify any differences associated with recurrent infections. The overall distribution of B cell populations was significantly different between those with recurrent infections and those without (Chi square *p* < 0.0001). The lowest numbers and percentage of CSM B cells occurred in the recurrent infection subcohort of the patients with 22q11.2DS, however, this did not reach significance using the Wilcoxon Rank Sum Test (percentages are given in Fig. 1C, cell counts defined by scRNA-seq are given in Fig. 1D).

From these studies, we concluded that the overall distribution of B cell populations in 22q11.2DS was significantly altered compared to healthy donors. The subcohort with 22q11.2DS and recurrent infections had the lowest CSM B cells suggesting that this group might have evidence of impaired T cell help delivered by Tfh cells. We had previously identified normal B cell activation in vitro, supporting the concept that intrinsic B cell function is intact [14]. B cells exhibited a diminished somatic hypermutation frequency in patients compared to controls, suggesting Tfh dysfunction [14]. We therefore next examined the T cell compartment.

### T Cell Analysis

**Fig. 2:**
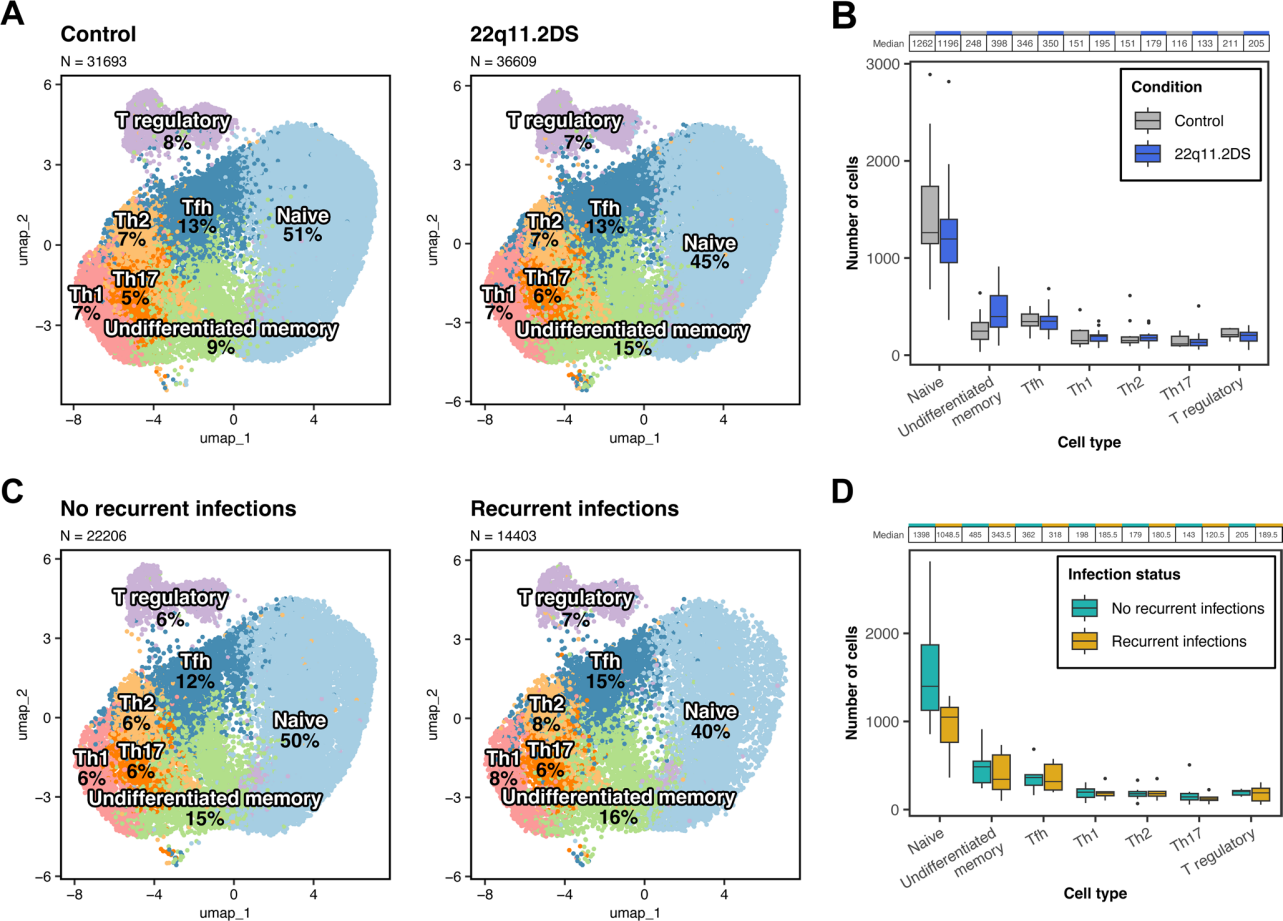
CD4 T cell populations. 2**A**) CD4 T cell subsets were defined using the gene expression signatures indicated in Supplemental Table 2. The UMAP plots demonstrate a shift away from naïve T cells and towards memory T cells in 22q11.2DS. This panel displays populations as percentages of the total CD4 cell population. 2**B**) Box plots comparing naïve and memory CD4 T cell counts between patients and controls. Numbers of cells defined by scRNA-seq are reported on the y-axis. The horizontal line in the box and whisker plots represents the median for the population. The median is listed above the graph. 2**C**) We specifically evaluated polarized CD4 memory T cells in the patients with 22q11.2DS +/- recurrent infections. Patients with recurrent infections had higher Tfh cells along with generally higher memory populations and lower naïve T cells. The display is as for panel (A) 2**D**) Box plots comparing polarized memory CD4 T cell numbers between patients with recurrent infections and those without. The display is as for panel (B) 2E) We utilized flow cytometry to confirm the increased Tfh cells identified through scRNA-seq as CD3 + CD4 + CXCR5 + ICOS+. 16 patients were compared with 16 age-matched controls. The fraction of Tfh cells within the CD4 compartment is displayed. **** indicates *p* < 0.0001. The ages of the patients with 22q11.2DS ranged from 10–39 with a mean of 16.7 years. The ages of the controls ranged from 9–38 with a mean of 19.7 years. The patients were 56% male. The controls were 44% male. The race and ethnicity for the controls was not recorded

T cells are the quintessential cell impacted by thymic hypoplasia in patients with the deletion [23, 32, 39]. We therefore assessed populations of CD4 T cells. The overall distribution of T cell subsets was altered in 22q11.2DS (Chi square *p* < 0.0001) with the altered distribution being reflective of a lower percentage naïve T cells in 22q11.2DS than healthy donors and higher memory T cells (percentages are given in Fig. 2A, cell counts defined by scRNA-seq are given in Fig. 2B). Individual population comparisons did not reach statistical significance. These data align with generally accepted T cell features in 22q11.2DS defined using flow cytometry and provide confidence in this dataset [20, 37, 57]. T cell subsets and maturation vary by age and we therefore analyzed our data using the age brackets in Table 1 and found no difference.

When we analyzed the subcohort with recurrent infections compared to the subcohort without recurrent infections, the overall distribution was significantly different between these subcohorts (Chi square *p* < 0.0001) with higher percentage of memory T cells in the group with recurrent infections (percentages are given in Fig. 2C, cell counts defined by scRNA-seq are given in Fig. 2D). Analysis of individual populations using the Wilcoxon Rank Sum test did not identify any single population that reached significance.

We additionally identified expression of *PECAM*, usually identified through flow cytometry analysis of CD31 for recent thymic immigrants in naïve CD4 T cells. *PECAM* expression was largely limited to naïve CD4 T cells, as expected. Within naive CD4 T cells, *PECAM* expression was far lower in patients (average log2 fold change of -0.2485) compared to controls with *p* = 4.3 × 10^− 12^. This is consistent with our understanding of the mechanism of disease. There was no difference between the patients with recurrent infection and those without recurrent infection, however.

We previously identified lower levels of somatic hypermutation in patients with 22q11.2DS, a specific marker for impaired T cell help [14]. Paradoxically, in our previous work, CD4/CXCR5/ICOS Tfh were higher in patients than controls. Activated Tfh defined by CCR7lo/PD1hi were also increased in 22q11.2DS [14]. To better understand any possible mechanisms of dysfunction, we therefore analyzed Tfh cells in 22q11.2DS overall using scRNA-seq. The highest levels of Tfh were noted in the subcohort with recurrent infections although without reaching significance using the Wilcoxon test (Fig. 2C and D). To understand if the Tfh were fundamentally altered, we compared BCL6 and IL21 expression in patients with 22q11.2DS and controls. There was no difference in expression (not shown). To validate the increased Tfh using a more traditional approach for the definition of Tfh, we performed flow cytometric analysis on a different cohort of patients and controls. Using flow cytometry, significantly higher levels of Tfh cells were seen in patients with 22q11.2DS compared to controls with *p* < 0.0001 (Fig. 2E). This replication cohort was not stratified for infection.

The low CSM B cells despite the high Tfh suggested possible Tfh cell dysfunction manifesting as compromised B cell help. The strength of scRNA-seq is the ability to identify changes in gene expression that might be associated with functional changes in cell populations. Therefore, we next analyzed RNA expression signatures.

### Differential Gene Expression and Pathway Analysis

**Fig. 3:**
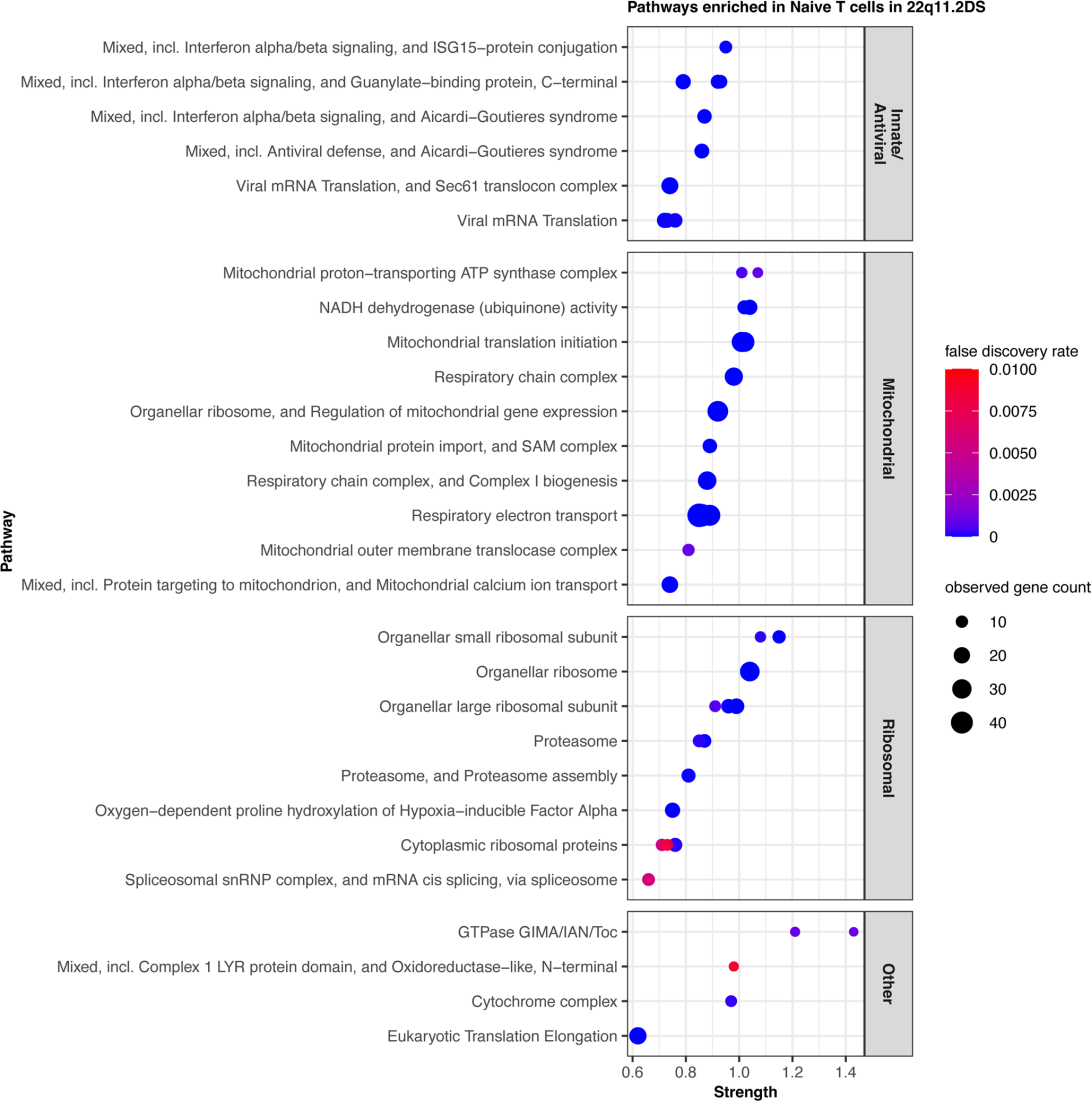
Differentially expressed genes within the naïve CD4 T cell population. We displayed STRING clusters according to Strength (x-axis), FDR (red-blue), and gene count (size of the circles) within the module. The Strength metric assesses the enrichment of that module and is defined as log10 (observed/expected). It represents a confidence score with high confidence described by values 0.7-1.0. All pathways shown had FDR < 0.01. The largest blue circles are therefore the STRING clusters with the highest gene count within the STRING cluster, and blue color indicates pathways with the lowest FDR. We divided significant STRING clusters according to cellular function, collapsing related STRING clusters into innate anti-viral, mitochondrial, ribosomal, and “other” groups. The full term names and ID numbers are given in Supplemental Table 3

The T cells are central to the pathogenesis of 22q11.2DS as we understand it [50]. Previous studies have demonstrated altered function by various methods ranging from proliferation to cytokine production and chromatin conformation [20, 29, 57, 75]. A dot plot presenting the top 10 gene expression differences in each cell type, comparing controls and 22q11.2DS, demonstrates widespread differences although ribosome genes dominate the picture suggesting proliferative differences (Supplemental Fig. 4). To further investigate T cells, we examined differentially expressed genes (DEGs) in naïve CD4 T cells using the Search Tool for the Retrieval of Interacting Genes (STRING) to identify a more nuanced picture of the pathways with differences in gene expression to avoid the dominance of the highly expressed ribosomal genes [66] (Fig. 3). We divided our DEGs into several categories. Multiple innate anti-viral pathways had a low FDR as did many mitochondrial STRING clusters. A number of ribosomal and splicing pathways also scored low FDRs in this analysis. The full names and ID numbers for all the identified pathways are given in Supplemental Table 3. Filtering out cells with high mitochondrial gene expression is typically used in the preprocessing stage of scRNA-seq analysis. To ensure the mitochondrial pathways represented true biology and not high cell death, we manually examined the individual samples for mitochondrial markers of cell death. There was no difference between patients and controls. This supports the mitochondrial expression signature observed in this analysis as related to the biologic attributes of the cell rather than non-specific cell death.

**Fig. 4:**
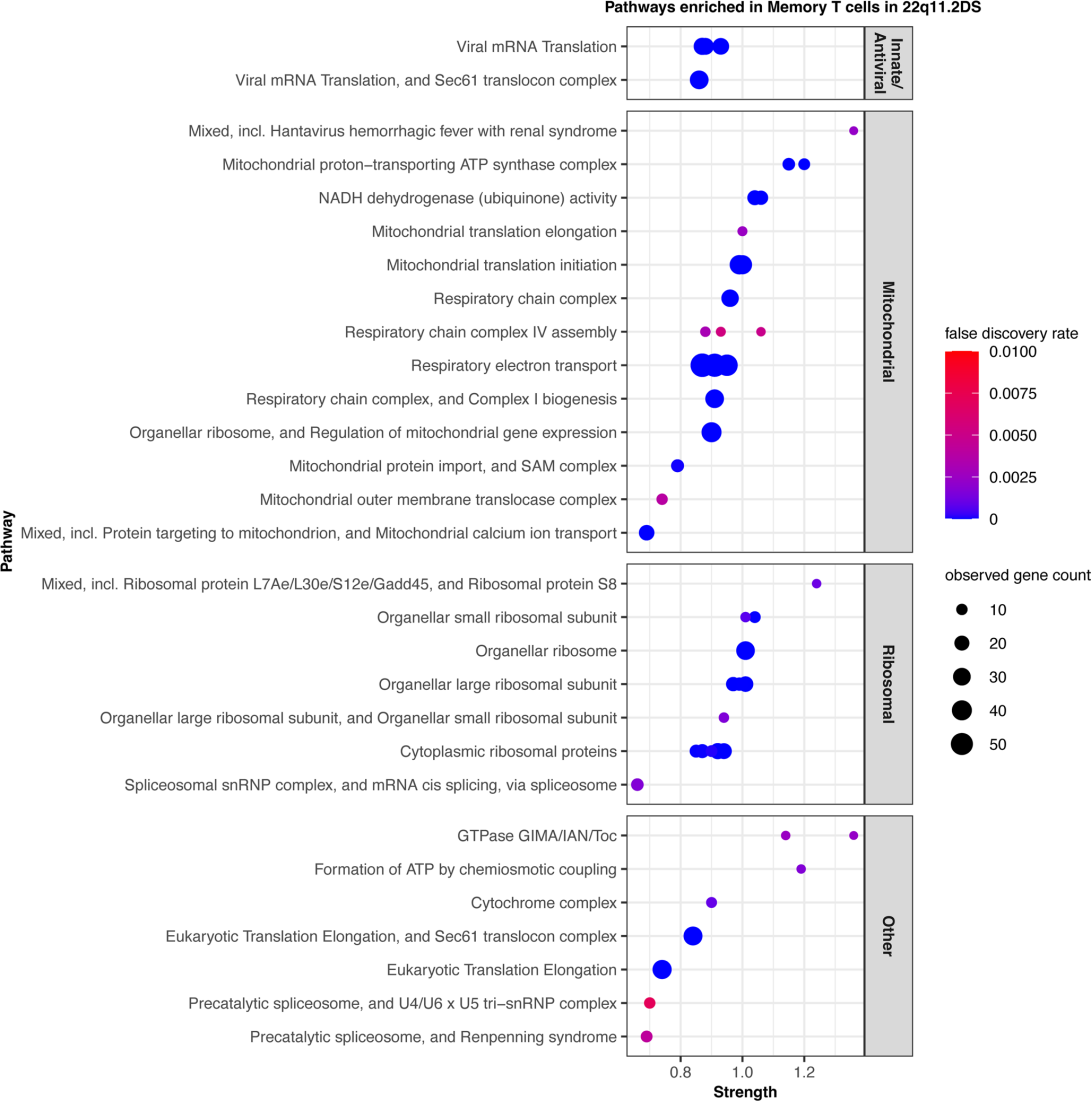
Differentially expressed genes within the memory CD4 T cell population. STRING clusters are displayed according to the schema described in the legend to Fig. 3. In memory CD4 T cells, the innate anti-viral STRING clusters are still represented, however, the strongest signals are from the mitochondrial and ribosomal pathways. All pathways shown had FDR < 0.01. The full term names and ID numbers are given in Supplemental Table 4

To distinguish effects related to antigen exposure, we similarly examined the CD4 memory T cells. These cells also had strong mitochondrial, ribosome, and anti-viral STRING clusters with low FDRs (Fig. 4). These data collectively defined a T cell compartment with a highly altered phenotype as defined by scRNA-seq. The strong mitochondrial signature and innate anti-viral signature in both memory and naïve CD4 T cells implied a significant effect from either the external milieu or from T cell intrinsic dysfunction.

In spite of increased Tfh cells, patients have been described as having diminished somatic hypermutation and CSM B cell counts [14, 30, 31, 52, 74]. We hypothesized that the Tfh cells similarly had an altered phenotype that might impact function and we examined the scRNA-seq data to better understand the phenotype as defined by DEGs.

**Fig. 5:**
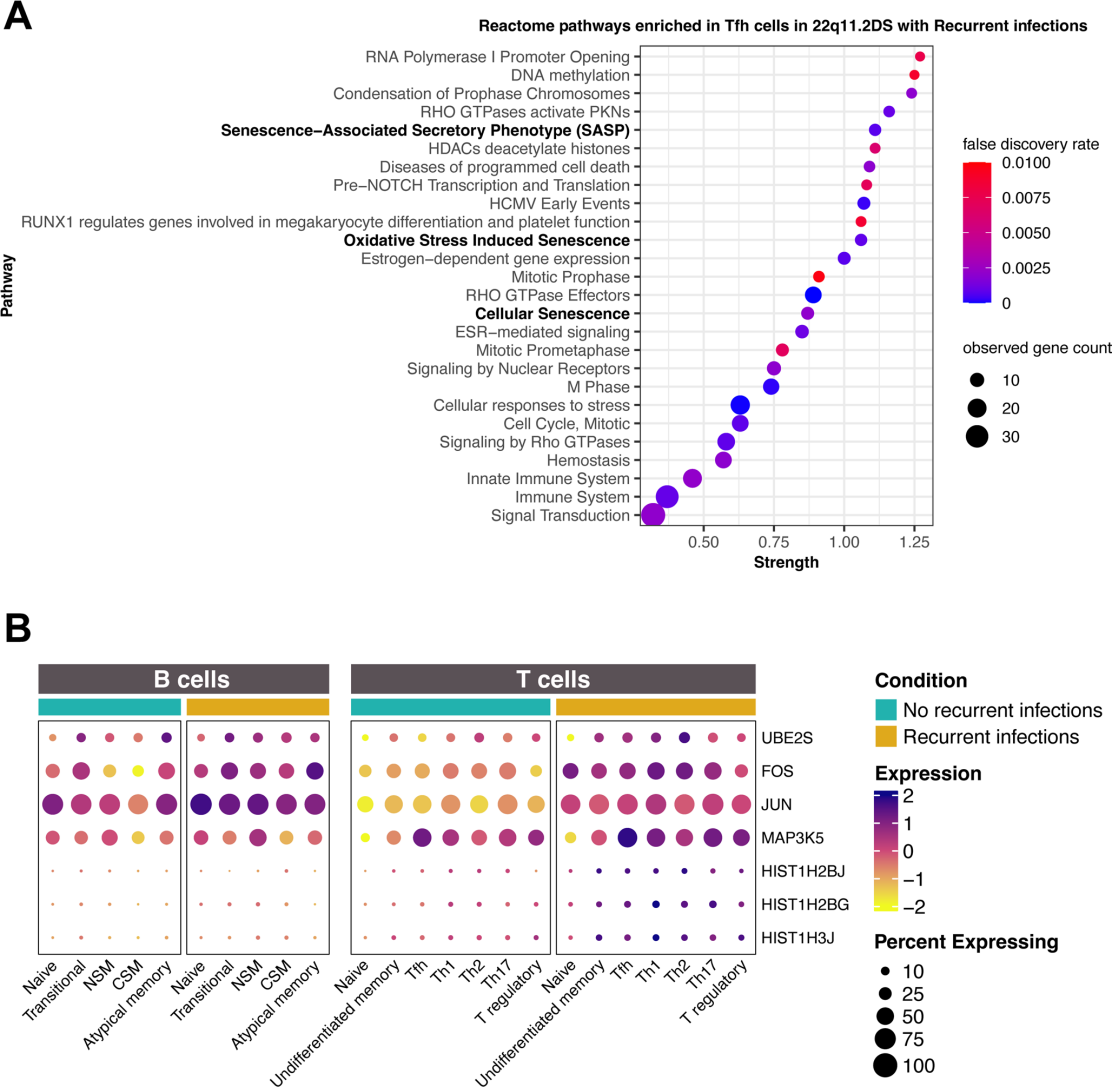
Differentially expressed modules within the Tfh cells. 5**A**) The Reactome pathways are displayed comparing DEGs in Tfh in patients with recurrent infection vs. those without. The display schema is the same as in Fig. 3. We noted three senescence pathways (bolded). All pathways shown had FDR < 0.01. The full term names and ID numbers are given in Supplemental Table 5. 5**B**) We identified the common genes between these three senescence pathways and portrayed the expression according to whether the patients had a history of recurrent infections. In this dotplot, the highest expression levels were seen in the patients with recurrent infections (blue bar) across all T cell subsets. The effect was gene specific in the B cell populations. In this display, the greatest increase in expression is denoted as purple on the yellow-purple color scale (where is expression is scaled across all groups within each gene) and the percentage of cells exhibiting increased expression is denoted by the size of the circle

The Tfh cells in 22q11.2DS with recurrent infections had a variety of enriched modules unique to this cell type (Fig. 5A). Overall, these pathways were different than those seen in other memory CD4 T cells. Embedded within the pathway analysis were three distinct senescence pathways (Fig. 5A bolded). We focused on these senescence modules within the Tfh when comparing patients with recurrent infections vs. those without recurrent infections. These three senescence pathways (Reactome pathways HSA-2559582, HSA-2559580, HSA-2559583 with FDR = 0.00072–0.0021) were strongly enriched in the patients with the lowest CSM B cells and recurrent infections. We identified which of the DEGs were found in all three the of the senescence pathways and among those common genes, we examined expression of these genes across all T and B cell subsets and portrayed them in a dot plot (Fig. 5B). Indeed, across all T cell types, we saw increased expression of the central senescence markers, specifically highest in those with recurrent infections. The naïve T cells from the patients without recurrent infections had the lowest expression overall for this signature. *MAP3K5* was expressed in all polarized cell types at higher levels than in naïve T cells. This member of the MAP kinase pathway activates AP-1, a known regulator of senescence [45, 54].

**Fig. 6:**
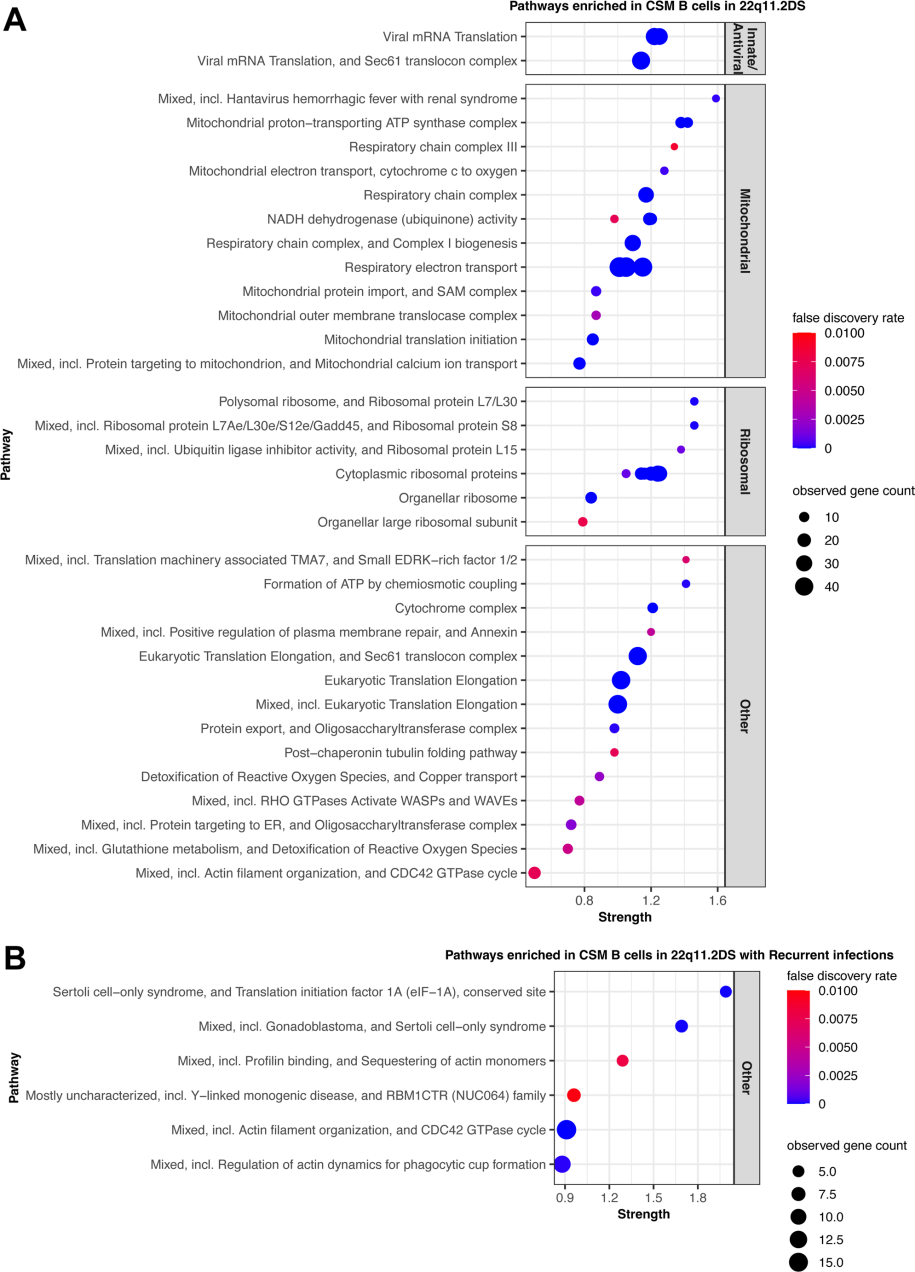
Differentially expressed genes within the class switched memory (CSM) B cell population. 6**A**) STRING pathways are displayed that are enriched in CSM B cell population in 22q11.2DS syndrome. Concordant with the T cells, antiviral, mitochondrial, and ribosomal signatures dominate the DEGs. All pathways shown had FDR < 0.01. The full term names and ID numbers are given in Supplemental Table 6. 6**B**) STRING pathways are displayed that are enriched in the subcohort of patients with recurrent infections. All pathways shown had FDR < 0.01. The full term names and ID numbers are given in Supplemental Table 7

Having observed altered Tfh cells, we reanalyzed our B cell scRNA-seq data to define DEGs within the B cell compartment. We initially analyzed the data as a dot plot to portray differences in each B cell subset between 22q11.2DS and controls (Supplemental Fig. 5). As was true for T cells, the ribosomal genes dominated the picture due to their high level of expression and their dynamic expression with proliferation. We therefore utilized the STRING display to better capture the landscape of pathways impacted by DEGs. The CSM B cells exhibited conceptually very similar DEGs to the T cells (Fig. 6A and Supplemental Fig. 6). Concordant with the T cell analysis, B cells exhibited an anti-viral signature, mitochondrial signature, and a ribosome signature. The OXPHOS upregulation seen in the mitochondrial signature is characteristic of proinflammatory B cells and early activation stages [36, 59]. A comparable signature has been observed in B cells from patients with SLE [61, 67]. We also noted that the senescence signature identified in patients with recurrent infection in Fig. 5B was also seen in B cells among those with recurrent infections. The effect size was smaller and the most differentially expressed gene was *FOS* rather than *MAP3K5*. Among CSM B cells, *JUN* also exhibited increased expression in the subcohort with recurrent infection. To further characterize the B cells, we examined the expression of *FCRL4* and *FCRL5*, two Fc-like receptors linked to atypical or anergic B cells [35]. Most *FCRL4* + cells express low levels of CD21 and may be optimized for tissues. *FCRL5* + atypical memory B cells correspond to the CD21lo cells seen in common variable immunodeficiency and chronic infection states [58, 60]. We did not detect significant expression of *FCRL4*. *FCRL5* was expressed primarily in the atypical memory B cells and was significantly higher in the patients with 22q11.2DS compared to controls (*p* = 3.1 × 10 − 5). It was also higher in the patients with recurrent infections compared to those without recurrent infections with *p* = 0.012. When we examined CSM B cells and compared DEGs between those with infection and those without recurrent infections, the individual modules did not align with senescence (Fig. 6B).

We considered several explanations for findings of senescence, mitochondrial gene expression, and innate immunity across cell types predicted to have both direct and indirect impact of 22q11.2 deletion. Although subjects were specifically recruited to be free of infection, we considered whether chronic infections in an immunocompromised condition might lead to changes. We performed an O-link analysis on plasma from the same donors as were used in the scRNA-seq analyses. There was no evidence of inflammation as defined by this specific analysis (Supplemental Fig. 6). Indeed, only 7 ligands had levels that were significantly different between patients with 22q11/2DS and controls and they were all lower in the patients. We considered whether our analytic approach enriched for these cell processes. As an alternative display of cell pathways, the Gene Ontology (GO) terms as “Biological Processes” were defined through DEGs. Those analyses revealed a similar set of pathways with mitochondria and viral processes identified in naïve T cells. For simplicity only the top 10 GO terms were displayed (Supplemental Fig. 7).

## Discussion

Diminished T cell numbers have been observed in 22q11.2DS since the first papers on DiGeorge syndrome and the cell type most impacted is naïve T cells. The diminished naïve T cells are a reflection of thymic hypoplasia, a common, but not invariant finding, in this syndrome. A central conundrum in 22q11.2DS is T cells counts are often normal in adulthood, yet there are limited clinical changes suggesting resolution of immunodeficiency [29, 57, 64]. In fact, for a subset of patients, autoimmunity, hypogammaglobulinemia, and even immune dysregulation dominates the clinical picture as the patients age [15, 29, 46, 55, 62, 65]. Various hypotheses have been developed to account for this observation. Possible contributors include T cell homeostatic pressure leading to dysfunction [20, 56, 57], T cell intrinsic defects related to the deletion [13], impaired regulatory T cells, intrinsic dysfunction of the B cells related to the deletion, and possibly disordered central tolerance in a setting with limited thymic volume [20, 42]. On a clinical basis, biomarkers of autoimmunity have been identified which suggest a central role for the T cells themselves [12, 15, 49]. Biomarkers for recurrent infections have received less attention but a recent study found low effector memory T cells in patients with recurrent infections [11].

Two critical findings emerged from this study. Among the T cells, we saw enrichment of DEGs related to mitochondria and anti-viral pathways typified by type I interferon signaling. Type I interferons have been highlighted in recent years as a component of immuno-aging [10]. They enhance DNA damage through complex mechanisms and are themselves induced by genomic instability via the STING pathway [22]. In aging, accumulation of nucleic acids from transposable elements and mitochondria drive type I interferon expression across multiple tissues [5, 38, 73]. In T cells specifically, type I interferons drive exhaustion and senescence via FOXO1 [19]. A feed forward loop whereby type I interferons contribute to mitochondrial dysfunction and destabilization of the mitochondrial genome leading to additional type I interferons, completes the model [27, 33]. The fact that the anti-viral-type I interferon signature and senescence was also seen in B cells, particularly in the patients with recurrent infections, supports a contribution from the milieu. In systemic lupus erythematosus, type I interferons have been shown to activate Tfh cells and drive increased IL-21 expression, possibly contributing to defective peripheral tolerance of B cells [17]. Mitochondrial dysfunction has been observed in neuronal cells and may be an intrinsic feature of the syndrome [34, 68].

The humoral component of the immunodeficiency has been recognized for many years [24] but only a small subset of patients with low CSM B cells are thought to evolve hypogammaglobulinemia [14, 55]. Therefore, there has been a gap in knowledge related to this finding and whether patients with low CSM B cells are on an evolving slide towards antibody dysfunction or whether the finding can be static and medically inconsequential. Our cross-sectional study cannot answer long term outcome questions, however, our data provides an improved understanding of the features found in patients with recurrent infections and supports a model where patients with recurrent infection have the most altered T and B cell compartments with expression of markers of senescence. Senescence is clinically associated with infection in other settings [40, 44], which supports this as a medically important observation.

This study focused on infections, an aspect recognized by clinicians yet poorly understood. In one study, 38% of patients experienced recurrent pneumonia and 6% of deaths in adults with 22q112DS were from pneumonia [4, 69]. In this study we examined immunologic correlates of infection, recognizing that anatomy, exposures, allergies, and medical co-morbidities influence infection risk and severity [25]. Another limitation of our analysis is that we examined Tfh in peripheral blood whereas their main site of action is in lymphoid tissue. Future studies of tonsillar tissue might provide further insights. However, it would be expected that the type I interferon effect would not be limited to circulating Tfh cells. Patients were recruited at a time when they did not clinically have an infection although we cannot rule out that some patients had a subclinical infection that might have contributed to the interferon signature. This would not have led to senescence, however. Additional limitations include a small sample size and lack of longitudinal assessments. The small sample size and variability in cell populations between samples may have contributed to the lack of statistical significance when comparing individual populations. Nevertheless, this is a robust scRNA-seq data set which has advanced our understanding of the alterations in both T cells and B cells in 22q11.2DS. This technology was instrumental in identifying a wide-spread effect on lymphocytes.

In summary, patients with 22q11.2 DS syndrome and recurrent infections had an increased proportion of Tfh cells with evidence of senescence. We propose that these features compromise class switching in B cells and contribute to altered CSM B cell maturation or survival with subsequent evolution of hypogammaglobulinemia in a subset of patients. Further longitudinal analyses will be required to test this hypothesis. For the moment, we conclude that 22q11.2DS exhibits not only an overall difference in the distribution of T cell subtypes compared to control, but also highly altered T cell phenotypes as defined by DEGs. We had previously identified a highly altered epigenome in 22q11.2DS which may underlie the altered gene expression. In that study, a type I interferon signature and markers of senescence were also identified [75]. Aligned with the senescence signature in T cells, is a B cell signature similarly reflective of impacts from type I interferons and senescence, albeit with a smaller effect size. Importantly, many of the features identified such as higher CD21lo B cells and low CSM B cells align with our current understanding of common variable immunodeficiency [8, 51, 60]. Whether this reflects any commonalities in mechanisms remains to be seen [51, 71].

The identification of senescence signatures in multiple lineages suggests that there may be a milieu affecting multiple cell types. Practical applications of this work include a greater attention to the group with recurrent infections as a sign of immune dysfunction and a recognition that the senescence effect could continue to progress with age.

## Supplementary Information

Below is the link to the electronic supplementary material.

Supplementary Material 1 (DOCX 6.50 MB)

## Data Availability

The datasets generated during and/or analyzed during the current study are available from the corresponding author on reasonable request.
