# Supplementary material for: Follicular Helper T Cells and B Cell Maturation in Patients with 22q11.2 Deletion Syndrome and Recurrent Infections
Source: J Clin Immunol. 2026 Feb 3;46(1):20. doi: 10.1007/s10875-026-01987-2 (PMC12913360; doi:10.1007/s10875-026-01987-2)
Supplement: Supplementary file 1 — Supplementary Material 1 (DOCX 6.50 MB) [file 10875_2026_1987_MOESM1_ESM.docx]

**Supplemental Methods and Data**

**Tables**

**Supplemental Table 1:** Clinical data relating to patients

**Supplemental Table 2:** B cell and T cell markers used in scRNA-seq

**Supplemental Table 3:** Naïve T cell Patient v. Control Local Network Cluster pathway results from STRINGdb, as shown in Figure 3.

**Supplemental Table 4:** Memory T cell Patient v. Control Local Network Cluster pathway results from STRINGdb, as shown in Figure 4.

**Supplemental Table 5:** Tfh Recurrent Infection v. No recurrent infection Reactome pathway results from STRINGdb, as shown in Figure 5A.

**Supplemental Table 6:** CSM B cells Patient v. Control Local Network Cluster pathway results from STRINGdb, as shown in Figure 6A.

**Supplemental Table 7:** CSM B cells Infection v. No recurrent infection Local Network Cluster pathway results from STRINGdb, as shown in Figure 6B.

**Figures**

**Supplemental Figure 1:** B cell and T cell specific marker gene expression displayed as a heat map.

**Supplemental Figure 2:** Violin plot of B cell specific marker genes from Supplemental Table 2.

**Supplemental Figure 3**: Violin plot of T cell specific marker genes from Supplemental Table 2.

**Supplemental Figure 4:** Dot plot demonstration of gene expression differences in 22q11.2DS compared to control. The top 10 genes for each T cell type are displayed.

**Supplemental Figure 5:** Dot plot demonstration of gene expression differences in 22q11.2DS compared to control. The top 10 genes for each B cell type are displayed.

**Supplemental Methods**

**References**

**Supplemental Data**

**Supplemental Table 1**

Clinical data relating to patients

| **ID** | **Dx** | **Sex** | **Age** | **Race** | **Age bracket** | **Main clinical features** | **Cardiac surgery** | **Recurrent infections status** | **Infections details** | **Autoimmunity** | **Atopy** | **IgG** | **IgA** | **IgM** | **CD3** | **CD4** | **CD19** | **NK** |
| --- | --- | --- | --- | --- | --- | --- | --- | --- | --- | --- | --- | --- | --- | --- | --- | --- | --- | --- |
| **C11662A** | CH22 | Female | 38 | Black | 17 years and older | Learning differences | No | No | OM: 0  Pneumonia: 0  Sinusitis: 0  UTI: 1 | no | no | 1240 | 353 | 121 | 1069 | 666 | 554 | 326 |
| **C11923A** | CH22 | Male | 11 | Black | 11-16 years | Imperforate anus, VSD | No | No | OM: 2  Pneumonia: 0  Sinusitis: 0  UTI: 0 | no | yes | 1090 | 126 | 50 | 1752 | 1140 | 561 | 207 |
| **C12035A** | CH22 | Male | 10 | White | 5-10 years | Polydactyly, Aberrant right subclavian, hearing loss | No | No | OM: 4  Pneumonia: 0  Sinusitis: 4  UTI: 0 | no | yes | 1010 | 154 | 48 | 845 | 376 | 333 | 494 |
| **C12131A** | CH22 | Male | 13 | White | 11-16 years | VACTERL, VSD, Tethered cord | No | No | OM: 0  Pneumonia: 0  Sinusitis: 0  UTI: 1 | no | yes | 744 | 217 | 50 | 2871 | 1883 | 456 | 104 |
| **C12389A** | CH22 | Male | 41 | White | 17 years and older | Diabetes, cleft palpate, hypothyroid | No | No | OM: 2  Pneumonia: 0  Sinusitis: 0  UTI: 0  Warts | no | no | 955 | 49 | 25 | 886 | 489 | 144 | 96 |
| **C12670A** | CH22 | Female | 10 | Other | 5-10 years | ASD, hypocalcemia, scoliosis | Yes | No | OM: 3  Pneumonia: 0  Sinusitis: 1  UTI: 0 | no | no | 742 | 36 | 64 | 1721 | 1103 | 512 | 276 |
| **C12671A** | CH22 | Female | 8 | Black | 5-10 years | Seizures, ASD, polydactyly | No | No | OM: 0  Pneumonia: 0  Sinusitis: 0  UTI: 0 | no | yes | 1070 | 257 | 164 | 3057 | 1425 | 849 | 195 |
| **C11758A** | CH22 | Female | 15 | White | 11-16 years | VSD, hypocalcemia | Yes | Yes | OM: 8  Pneumonia: 0  Sinusitis: 10  UTI: 0  PE tubes | no | no | 1120 | 118 | 57 | 982 | 710 | 390 | 230 |
| **C11796A** | CH22 | Male | 13 | Black | 11-16 years | Learning differences | No | Yes | OM: TNC  Pneumonia: 2  Sinusitis: 1  UTI: 0  PE tubes | no | yes | 811 | 65 | 25 | 1628 | 1333 | 803 | 973 |
| **C12061A** | CH22 | Male | 8 | White | 5-10 years | Club feet, PDA, laryngomalacia | No | Yes | OM: 8  Pneumonia: 2  Sinusitis: 0  UTI: 0  Candida | no | yes | 959 | 210 | 34 | 999 | 564 | 487 | 353 |
| **C12172A** | CH22 | Female | 8 | White | 5-10 years | Premature, Hypotonia, PFO | No | Yes | OM: 6  Pneumonia: 0  Sinusitis: 10  UTI: 0  CVID  PE tubes | no | yes | 526 | 90 | 44 | 1873 | 963 | 817 | 389 |
| **C12334A** | CH22 | Female | 15 | White | 11-16 years | Hypocalcemia, hearing loss | No | Yes | OM: TNC  Pneumonia: 0  Sinusitis: 0  UTI: TNC  PE tubes | no | Yes | 1510 | 221 | 112 | 655 | 286 | 83 | 467 |
| **C12686A** | CH22 | Male | 9 | White | 5-10 years | Hearing loss, laryngomalacia | No | Yes | OM: 6  Pneumonia: 0  Sinusitis: 6  UTI: 0  SAD | no | no | 852 | 275 | 20 | 1281 | 682 | 393 | 412 |

ASD= atrial septal defect, PE=pressure equalization, PFO=patent foramen ovale, ROM= recurrent otitis media, SAD= specific antibody deficiency, UTI= urinary tract infection, VACTERL= vertebral defects, anal atresia, cardiac defects, tracheo-esophageal fistula, renal anomalies, and limb abnormalities, VSD= Ventriculoseptal defect. Infections: OM= otitis media, PE tubes= pressure equalization tubes, UTI= urinary tract infection, SAD= specific antibody deficiency, CVID= common variable immunodeficiency. Definition of recurrent infections: >5OM or >2 pneumonias or > 4 sinus infections plus any other infections. UTI was not counted towards recurrent infection. TNC=too numerous to count.

**Supplemental Table 2**

B cell and T cell markers used in scRNA-seq

| **UMAPs** | **Cell type** | **Cell subtype** | **Gene markers up** | **Gene markers down** |
| --- | --- | --- | --- | --- |
| **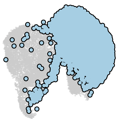** | **B cell** | **Naïve** | **CD19, CD20 (MS4A1), IGHM, IGHD** |  |
| **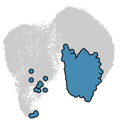** | **B cell** | **Transitional** | **CD19, CD20 (MS4A1), IGHM, IGHD** | **CD27** |
| **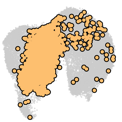** | **B cell** | **Non-switched memory (IgM)** | **CD19, CD20 (MS4A1), IGHM, IGHD, CD27** |  |
| **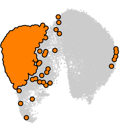** | **B cell** | **Class-switched memory (IgG)** | **CD19, CD20 (MS4A1), CD27, CD38, SDC1, IGHG1, IGHG2, IGHG3, IGHA** | **IGHM, IGHD** |
| **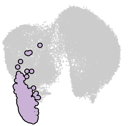** | **B cell** | **Atypical memory** | **CD19, CD20 (MS4A1), FCRL5, FCRL4** | **CD27** |
|  |  |  |  |  |
| **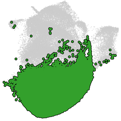** | **T cell** | **Naïve** | **CD3D, CD3E, CD4, IL7R, CCR7** |  |
| **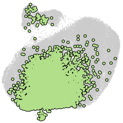** | **T cell** | **Undifferentiated memory** | **CD3D, CD3E, CD4, S100A4, w/o other significant TF expression** |  |
| **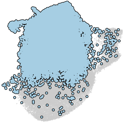** | **T cell** | **Memory** | **CD3D, CD3E, CD4, IL7R, S100A4** |  |
| **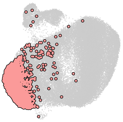** | **T cell** | **Th1** | **CD3D, CD3E, CD4, STAT1, STAT4, TBX21, CXCR6, CXCR3, TCF7, IL12RB1, IL12RB2, IFNGR1, IFNGR2, IFNG, IL2, TNF, LTA** |  |
| **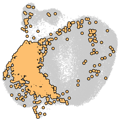** | **T cell** | **Th2** | **CD3D, CD3E, CD4, GATA3, STAT6, PTGDR2, IL4R, IL5, IL13, CCR4, IL17RB, MAF, IL10** |  |
| **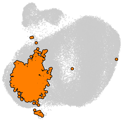** | **T cell** | **Th17** | **CD3D, CD3E, CD4, IL23R, CCR6, IL1R1, IL1RAP, RORA, RORC, STAT3, CCL20** |  |
| **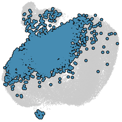** | **T cell** | **Tfh** | **CD3D, CD3E, CD4, ICOS, CXCR5, BCL6, PDCD1, IL21** |  |
| **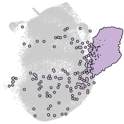** | **T cell** | **Treg** | **CD3D, CD3E, CD4, FOXP3, CTLA4, IL2RA, TIGIT, IL7R** |  |

**Supplemental Table 3**

Naïve T cell Patient v. Control Local Network Cluster pathway results from STRINGdb, as shown in Figure 3.

| **#term ID** | **term description** | **observed gene count** | **background gene count** | **strength** | **false discovery rate** |
| --- | --- | --- | --- | --- | --- |
| CL:165 | Cytoplasmic ribosomal proteins | 13 | 61 | 0.76 | 0.00018 |
| CL:3706 | Proteasome | 11 | 42 | 0.85 | 0.0002 |
| CL:671 | Organellar small ribosomal subunit | 8 | 18 | 1.08 | 0.0002 |
| CL:11166 | Cytochrome complex | 9 | 26 | 0.97 | 0.00024 |
| CL:11266 | Mitochondrial proton-transporting ATP synthase complex | 8 | 21 | 1.01 | 0.00048 |
| CL:572 | Organellar large ribosomal subunit | 9 | 30 | 0.91 | 0.00061 |
| CL:11268 | Mitochondrial proton-transporting ATP synthase complex | 7 | 16 | 1.07 | 0.00089 |
| CL:29797 | GTPase GIMA/IAN/Toc | 6 | 10 | 1.21 | 0.00097 |
| CL:29801 | GTPase GIMA/IAN/Toc | 5 | 5 | 1.43 | 0.00099 |
| CL:11526 | Mitochondrial outer membrane translocase complex, and TIM23 mitochondrial import inner membrane translocase complex | 10 | 42 | 0.81 | 0.001 |
| CL:166 | Cytoplasmic ribosomal proteins | 10 | 53 | 0.71 | 0.0056 |
| CL:2036 | Spliceosomal snRNP complex, and mRNA cis splicing, via spliceosome | 11 | 65 | 0.66 | 0.0058 |
| CL:167 | Cytoplasmic ribosomal proteins | 9 | 45 | 0.73 | 0.0083 |
| CL:11862 | Mixed, incl. Complex 1 LYR protein domain, and Oxidoreductase-like, N-terminal | 6 | 17 | 0.98 | 0.0088 |

**Supplemental Table 4**

Memory T cell Patient v. Control Local Network Cluster pathway results from STRINGdb, as shown in Figure 4.

| **#term ID** | **term description** | **observed gene count** | **background gene count** | **strength** | **false discovery rate** |
| --- | --- | --- | --- | --- | --- |
| CL:169 | Cytoplasmic ribosomal proteins | 11 | 32 | 0.9 | 0.00011 |
| CL:671 | Organellar small ribosomal subunit | 8 | 18 | 1.01 | 0.00071 |
| CL:11166 | Cytochrome complex | 9 | 26 | 0.9 | 0.00095 |
| CL:271 | Mixed, incl. Ribosomal protein L7Ae/L30e/S12e/Gadd45, and Ribosomal protein S8 | 6 | 8 | 1.24 | 0.0011 |
| CL:573 | Organellar large ribosomal subunit, and Organellar small ribosomal subunit | 8 | 21 | 0.94 | 0.0016 |
| CL:11270 | Formation of ATP by chemiosmotic coupling | 6 | 9 | 1.19 | 0.0017 |
| CL:2036 | Spliceosomal snRNP complex, and mRNA cis splicing, via spliceosome | 13 | 65 | 0.66 | 0.0017 |
| CL:11137 | Mixed, incl. Hantavirus hemorrhagic fever with renal syndrome, and Ribosomal protein/NADH dehydrogenase domain | 5 | 5 | 1.36 | 0.0023 |
| CL:29801 | GTPase GIMA/IAN/Toc | 5 | 5 | 1.36 | 0.0023 |
| CL:29797 | GTPase GIMA/IAN/Toc | 6 | 10 | 1.14 | 0.0025 |
| CL:576 | Mitochondrial translation elongation | 7 | 16 | 1 | 0.0025 |
| CL:11330 | Respiratory chain complex IV assembly, and Mitochondrial splicing suppressor 51-like, C-terminal domain | 8 | 24 | 0.88 | 0.0031 |
| CL:11526 | Mitochondrial outer membrane translocase complex, and TIM23 mitochondrial import inner membrane translocase complex | 10 | 42 | 0.74 | 0.0039 |
| CL:2037 | Precatalytic spliceosome, and Renpenning syndrome | 11 | 52 | 0.69 | 0.0042 |
| CL:11334 | Respiratory chain complex IV assembly | 6 | 12 | 1.06 | 0.0049 |
| CL:11332 | Respiratory chain complex IV assembly | 7 | 19 | 0.93 | 0.0054 |
| CL:2038 | Precatalytic spliceosome, and U4/U6 x U5 tri-snRNP complex | 10 | 46 | 0.7 | 0.0071 |
| CL:167 | Cytoplasmic ribosomal proteins | 14 | 45 | 0.85 | 1.10E-05 |
| CL:569 | Organellar large ribosomal subunit | 15 | 35 | 0.99 | 1.31E-07 |
| CL:11420 | Mixed, incl. Protein targeting to mitochondrion, and Mitochondrial calcium ion transport | 22 | 104 | 0.69 | 1.33E-06 |
| CL:572 | Organellar large ribosomal subunit | 13 | 30 | 1 | 1.39E-06 |
| CL:567 | Organellar large ribosomal subunit | 22 | 50 | 1.01 | 1.42E-11 |
| CL:11070 | Respiratory chain complex, and Complex I biogenesis | 34 | 97 | 0.91 | 1.62E-15 |
| CL:168 | Cytoplasmic ribosomal proteins | 13 | 40 | 0.87 | 2.02E-05 |
| CL:118 | Eukaryotic Translation Elongation, and This family consists of several GAGE and XAGE proteins which are found exclusively in humans. The function of this family is unknown although they have been implicated in human cancers (PMID:11992404) | 36 | 150 | 0.74 | 2.11E-12 |
| CL:11065 | Respiratory electron transport, ATP synthesis by chemiosmotic coupling, and heat production by uncoupling proteins., and Cytochrome complex | 56 | 160 | 0.91 | 2.19E-25 |
| CL:140 | Eukaryotic Translation Elongation, and Sec61 translocon complex | 35 | 116 | 0.84 | 2.21E-14 |
| CL:143 | Viral mRNA Translation, and Sec61 translocon complex | 34 | 108 | 0.86 | 2.21E-14 |
| CL:11077 | Respiratory chain complex | 29 | 73 | 0.96 | 2.56E-14 |
| CL:558 | Organellar ribosome, and Regulation of mitochondrial gene expression | 40 | 115 | 0.9 | 2.67E-18 |
| CL:162 | Cytoplasmic ribosomal proteins | 24 | 66 | 0.92 | 2.84E-11 |
| CL:159 | Viral mRNA Translation | 26 | 71 | 0.93 | 3.24E-12 |
| CL:166 | Cytoplasmic ribosomal proteins | 17 | 53 | 0.87 | 3.65E-07 |
| CL:11061 | Respiratory electron transport, ATP synthesis by chemiosmotic coupling, and heat production by uncoupling proteins., and Cytochrome complex | 58 | 180 | 0.87 | 3.86E-25 |
| CL:11080 | NADH dehydrogenase (ubiquinone) activity | 18 | 36 | 1.06 | 4.01E-10 |
| CL:148 | Viral mRNA Translation | 28 | 87 | 0.87 | 4.15E-12 |
| CL:560 | Mitochondrial translation initiation | 37 | 87 | 0.99 | 4.23E-19 |
| CL:11069 | Respiratory electron transport, ATP synthesis by chemiosmotic coupling, and heat production by uncoupling proteins. | 47 | 121 | 0.95 | 4.46E-23 |
| CL:165 | Cytoplasmic ribosomal proteins | 23 | 61 | 0.94 | 4.59E-11 |
| CL:11524 | Mitochondrial protein import, and SAM complex | 14 | 52 | 0.79 | 4.65E-05 |
| CL:11079 | NADH dehydrogenase (ubiquinone) activity | 20 | 42 | 1.04 | 5.14E-11 |
| CL:11268 | Mitochondrial proton-transporting ATP synthase complex | 11 | 16 | 1.2 | 5.36E-07 |
| CL:568 | Organellar large ribosomal subunit | 18 | 44 | 0.97 | 5.42E-09 |
| CL:566 | Organellar ribosome | 34 | 77 | 1.01 | 5.43E-18 |
| CL:11266 | Mitochondrial proton-transporting ATP synthase complex | 13 | 21 | 1.15 | 5.74E-08 |
| CL:562 | Mitochondrial translation initiation | 36 | 82 | 1 | 5.76E-19 |
| CL:152 | Viral mRNA Translation | 27 | 81 | 0.88 | 5.77E-12 |
| CL:668 | Organellar small ribosomal subunit | 11 | 23 | 1.04 | 8.32E-06 |

**Supplemental Table 5**

Tfh Recurrent Infection v. No recurrent infection Reactome pathway results from STRINGdb, as shown in Figure 5A.

| **#term ID** | **term description** | **observed gene count** | **background gene count** | **strength** | **false discovery rate** |
| --- | --- | --- | --- | --- | --- |
| **HSA-73728** | **RNA Polymerase I Promoter Opening** | **4** | **32** | **1.27** | **0.0077** |
| **HSA-5334118** | **DNA methylation** | **4** | **34** | **1.25** | **0.0089** |
| **HSA-2299718** | **Condensation of Prophase Chromosomes** | **5** | **43** | **1.24** | **0.002** |
| **HSA-5625740** | **RHO GTPases activate PKNs** | **6** | **63** | **1.16** | **0.001** |
| **HSA-2559582** | **Senescence-Associated Secretory Phenotype (SASP)** | **7** | **81** | **1.11** | **0.00072** |
| **HSA-3214815** | **HDACs deacetylate histones** | **5** | **59** | **1.11** | **0.0062** |
| **HSA-9645723** | **Diseases of programmed cell death** | **6** | **73** | **1.09** | **0.002** |
| **HSA-1912408** | **Pre-NOTCH Transcription and Translation** | **5** | **62** | **1.08** | **0.0071** |
| **HSA-9609690** | **HCMV Early Events** | **8** | **102** | **1.07** | **0.00031** |
| **HSA-2559580** | **Oxidative Stress Induced Senescence** | **7** | **92** | **1.06** | **0.00085** |
| **HSA-8936459** | **RUNX1 regulates genes involved in megakaryocyte differentiation and platelet function** | **5** | **66** | **1.06** | **0.0087** |
| **HSA-9018519** | **Estrogen-dependent gene expression** | **8** | **119** | **1** | **0.00072** |
| **HSA-68875** | **Mitotic Prophase** | **6** | **111** | **0.91** | **0.0097** |
| **HSA-2559583** | **Cellular Senescence** | **8** | **163** | **0.87** | **0.0021** |
| **HSA-8939211** | **ESR-mediated signaling** | **9** | **190** | **0.85** | **0.0011** |
| **HSA-68877** | **Mitotic Prometaphase** | **8** | **201** | **0.78** | **0.0069** |
| **HSA-9006931** | **Signaling by Nuclear Receptors** | **10** | **265** | **0.75** | **0.002** |
| **HSA-68886** | **M Phase** | **14** | **382** | **0.74** | **0.00021** |
| **HSA-69278** | **Cell Cycle, Mitotic** | **15** | **526** | **0.63** | **0.00084** |
| **HSA-194315** | **Signaling by Rho GTPases** | **17** | **672** | **0.58** | **0.00084** |
| **HSA-109582** | **Hemostasis** | **15** | **607** | **0.57** | **0.002** |
| **HSA-168249** | **Innate Immune System** | **20** | **1041** | **0.46** | **0.0022** |
| **HSA-168256** | **Immune System** | **31** | **1979** | **0.37** | **0.00094** |
| **HSA-162582** | **Signal Transduction** | **35** | **2540** | **0.32** | **0.0021** |

**Supplemental Table 6**

CSM B cells Patient v. Control Local Network Cluster pathway results from STRINGdb, as shown in Figure 6A.

| #term ID | term description | observed gene count | background gene count | strength | false discovery rate |
| --- | --- | --- | --- | --- | --- |
| CL:11270 | Formation of ATP by chemiosmotic coupling | 6 | 9 | 1.41 | 0.00012 |
| CL:1625 | Protein export, and Oligosaccharyltransferase complex | 9 | 36 | 0.98 | 0.00017 |
| CL:11137 | Mixed, incl. Hantavirus hemorrhagic fever with renal syndrome, and Ribosomal protein/NADH dehydrogenase domain | 5 | 5 | 1.59 | 0.00024 |
| CL:11524 | Mitochondrial protein import, and SAM complex | 10 | 52 | 0.87 | 0.00033 |
| CL:11193 | Mitochondrial electron transport, cytochrome c to oxygen | 6 | 12 | 1.28 | 0.00037 |
| CL:170 | Cytoplasmic ribosomal proteins | 7 | 24 | 1.05 | 0.00097 |
| CL:217 | Mixed, incl. Ubiquitin ligase inhibitor activity, and Ribosomal protein L15 | 5 | 8 | 1.38 | 0.0011 |
| CL:1540 | Mixed, incl. Protein targeting to ER, and Oligosaccharyltransferase complex | 11 | 80 | 0.72 | 0.0018 |
| CL:9658 | Detoxification of Reactive Oxygen Species, and Copper transport | 8 | 40 | 0.89 | 0.0023 |
| CL:11526 | Mitochondrial outer membrane translocase complex, and TIM23 mitochondrial import inner membrane translocase complex | 8 | 42 | 0.87 | 0.003 |
| CL:19272 | Mixed, incl. Positive regulation of plasma membrane repair, and Annexin | 5 | 12 | 1.2 | 0.0043 |
| CL:17814 | Mixed, incl. RHO GTPases Activate WASPs and WAVEs, and ADF-H/Gelsolin-like domain superfamily | 9 | 59 | 0.77 | 0.0044 |
| CL:9657 | Mixed, incl. Glutathione metabolism, and Detoxification of Reactive Oxygen Species | 10 | 76 | 0.7 | 0.0052 |
| CL:3483 | Mixed, incl. Translation machinery associated TMA7, and Small EDRK-rich factor 1/2 | 4 | 6 | 1.41 | 0.0062 |
| CL:11082 | NADH dehydrogenase (ubiquinone) activity | 6 | 24 | 0.98 | 0.0072 |
| CL:17810 | Mixed, incl. Actin filament organization, and CDC42 GTPase cycle | 16 | 193 | 0.5 | 0.0072 |
| CL:26384 | Post-chaperonin tubulin folding pathway | 6 | 24 | 0.98 | 0.0072 |
| CL:567 | Organellar large ribosomal subunit | 8 | 50 | 0.79 | 0.0077 |
| CL:11170 | Respiratory chain complex III | 4 | 7 | 1.34 | 0.0087 |
| CL:166 | Cytoplasmic ribosomal proteins | 22 | 53 | 1.2 | 1.27E-15 |
| CL:11070 | Respiratory chain complex, and Complex I biogenesis | 31 | 97 | 1.09 | 1.33E-19 |
| CL:11266 | Mitochondrial proton-transporting ATP synthase complex | 13 | 21 | 1.38 | 1.42E-10 |
| CL:11166 | Cytochrome complex | 11 | 26 | 1.21 | 1.57E-07 |
| CL:152 | Viral mRNA Translation | 35 | 81 | 1.22 | 1.66E-25 |
| CL:162 | Cytoplasmic ribosomal proteins | 30 | 66 | 1.24 | 1.77E-22 |
| CL:11061 | Respiratory electron transport, ATP synthesis by chemiosmotic coupling, and heat production by uncoupling proteins., and Cytochrome complex | 48 | 180 | 1.01 | 1.94E-27 |
| CL:11080 | NADH dehydrogenase (ubiquinone) activity | 15 | 36 | 1.2 | 2.02E-10 |
| CL:11077 | Respiratory chain complex | 28 | 73 | 1.17 | 2.30E-19 |
| CL:167 | Cytoplasmic ribosomal proteins | 16 | 45 | 1.14 | 2.47E-10 |
| CL:562 | Mitochondrial translation initiation | 15 | 82 | 0.85 | 2.78E-06 |
| CL:11268 | Mitochondrial proton-transporting ATP synthase complex | 11 | 16 | 1.42 | 3.20E-09 |
| CL:140 | Eukaryotic Translation Elongation, and Sec61 translocon complex | 40 | 116 | 1.12 | 3.68E-26 |
| CL:143 | Viral mRNA Translation, and Sec61 translocon complex | 39 | 108 | 1.14 | 3.90E-26 |
| CL:11065 | Respiratory electron transport, ATP synthesis by chemiosmotic coupling, and heat production by uncoupling proteins., and Cytochrome complex | 47 | 160 | 1.05 | 4.17E-28 |
| CL:165 | Cytoplasmic ribosomal proteins | 28 | 61 | 1.25 | 5.13E-21 |
| CL:118 | Eukaryotic Translation Elongation, and This family consists of several GAGE and XAGE proteins which are found exclusively in humans. The function of this family is unknown although they have been implicated in human cancers (PMID:11992404) | 41 | 150 | 1.02 | 6.16E-24 |
| CL:168 | Cytoplasmic ribosomal proteins | 15 | 40 | 1.16 | 6.41E-10 |
| CL:11069 | Respiratory electron transport, ATP synthesis by chemiosmotic coupling, and heat production by uncoupling proteins. | 44 | 121 | 1.15 | 6.45E-29 |
| CL:256 | Polysomal ribosome, and Ribosomal protein L7/L30 | 6 | 8 | 1.46 | 7.61E-05 |
| CL:271 | Mixed, incl. Ribosomal protein L7Ae/L30e/S12e/Gadd45, and Ribosomal protein S8 | 6 | 8 | 1.46 | 7.61E-05 |
| CL:159 | Viral mRNA Translation | 33 | 71 | 1.25 | 7.61E-25 |
| CL:11420 | Mixed, incl. Protein targeting to mitochondrion, and Mitochondrial calcium ion transport | 16 | 104 | 0.77 | 7.74E-06 |
| CL:566 | Organellar ribosome | 14 | 77 | 0.84 | 8.10E-06 |
| CL:169 | Cytoplasmic ribosomal proteins | 12 | 32 | 1.16 | 8.60E-08 |
| CL:114 | Mixed, incl. Eukaryotic Translation Elongation, and This family consists of several GAGE and XAGE proteins which are found exclusively in humans. The function of this family is unknown although they have been implicated in human cancers (PMID:11992404) | 42 | 163 | 1 | 9.49E-24 |
| CL:11079 | NADH dehydrogenase (ubiquinone) activity | 17 | 42 | 1.19 | 9.87E-12 |

**Supplemental Table 7**

CSM B cells Infection v. No recurrent infection Local Network Cluster pathway results from STRINGdb, as shown in Figure 6B.

| **#term ID** | **term description** | **observed gene count** | **background gene count** | **strength** | **false discovery rate** |
| --- | --- | --- | --- | --- | --- |
| CL:17810 | Mixed, incl. Actin filament organization, and CDC42 GTPase cycle | 16 | 193 | 0.91 | 2.17E-06 |
| CL:7866 | Mixed, incl. Gonadoblastoma, and Sertoli cell-only syndrome | 6 | 12 | 1.69 | 4.01E-05 |
| CL:7869 | Sertoli cell-only syndrome, and Translation initiation factor 1A (eIF-1A), conserved site | 5 | 5 | 1.99 | 4.01E-05 |
| CL:17811 | Mixed, incl. Regulation of actin dynamics for phagocytic cup formation, and CDC42 GTPase cycle | 12 | 155 | 0.88 | 0.00017 |
| CL:17882 | Mixed, incl. Profilin binding, and Sequestering of actin monomers | 5 | 25 | 1.29 | 0.008 |
| CL:7827 | Mostly uncharacterized, incl. Y-linked monogenic disease, and RBM1CTR (NUC064) family | 7 | 74 | 0.96 | 0.0097 |

**Supplemental Figures**

**Supplemental Figure 1**

**A**

**
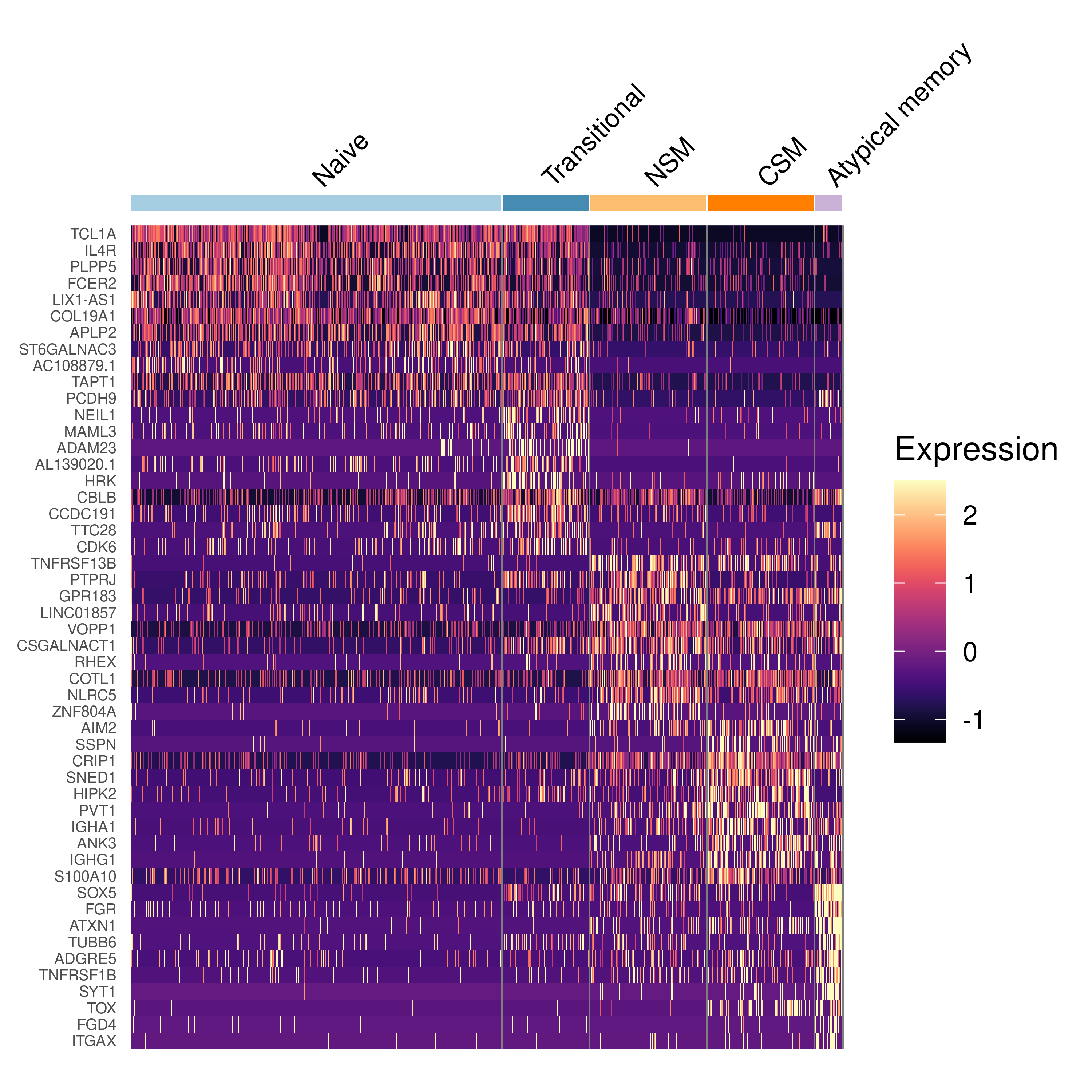
**

**B**

**Supplemental Figure 1**

1. The expression of the top 10 (by positive average log2 fold change) cell type specific marker genes are shown for each B cell subset. B) The expression of the top 10 (by positive average log2 fold change) cell type specific marker genes are shown for each T cell subset. For (B), the data was randomly downsampled to 14000 cells per condition (22q11.2DS and Controls) to improve performance in plotting.

**Supplemental Figure 2**

**Supplemental Figure 2**

The expression of the different B cell type specific marker genes is displayed as violin plots.

**Supplemental Figure 3**

**Supplemental Figure 3**

The expression of the different T cell type specific marker genes are displayed as violin plots.

**Supplemental Figure 4**

**Supplemental Figure 4.**

The expression of the top 10 (ordered by adjusted p-value) differentially expressed genes for each T cell type comparing 22q11.2DS and controls are presented as a dot plot. The top and center sections are dominated by ribosomal genes (RPS genes).

**Supplemental Figure 5**

**Supplemental Figure 5**

The expression of the top 10 (ordered by adjusted p-value) differentially expressed genes for each B cell type comparing 22q11.2DS and controls are presented as a dot plot. The top and center sections are dominated by ribosomal genes (RPS genes).

**Supplemental Figure 6**

**
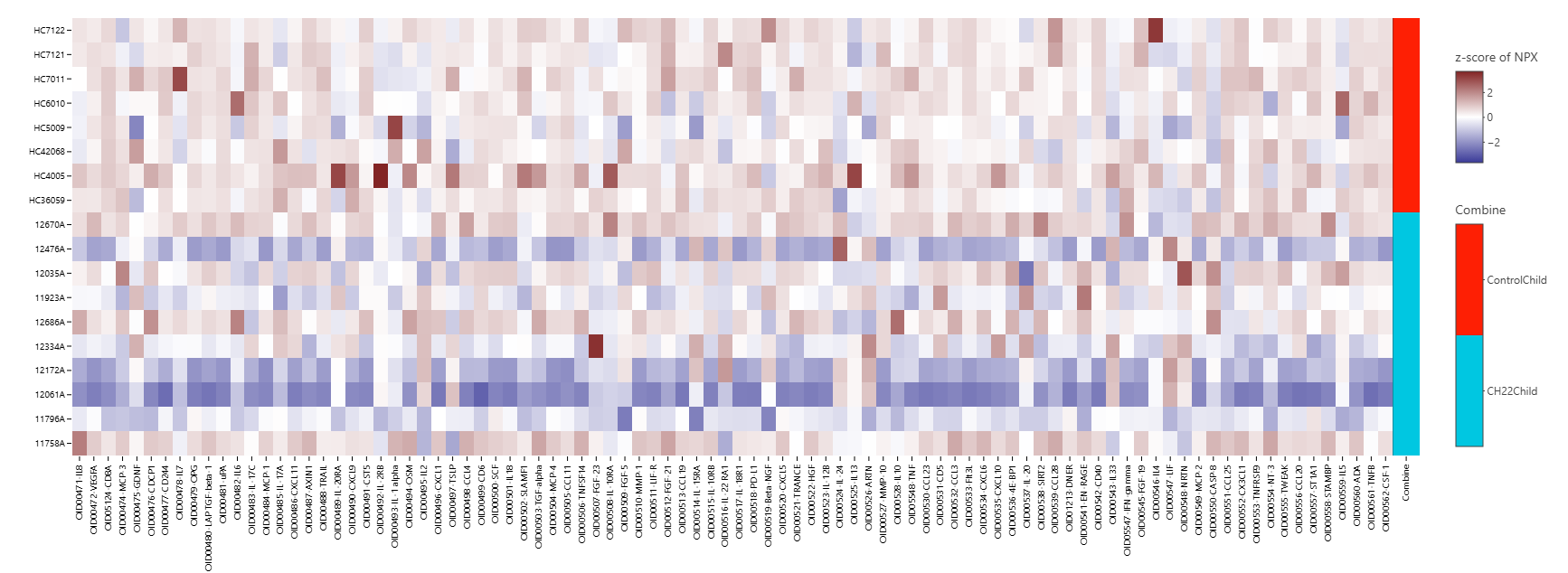
**

**Supplemental Figure 6**

This heat map displays levels of various inflammatory ligands detected by the Inflammation 96 O link panel. Not all subjects had plasma available, but the available subjects match the subjects in the scRNA-seq studies. Overall, levels of inflammatory ligands were lower in the patients (blue bar) compared to controls (red bar).

**Supplemental Figure 7**

**Supplemental Figure 7**

Enriched GO (Gene Ontology) “biological process” (BP) terms impacted by differentially expressed genes between patients and controls in each of the indicated cell types.

**Supplemental Methods**

*Pathway analysis*

STRINGdb^1^ (v12) was used to examine functional enrichment of differentially upregulated genes for specific cell types. Differential expression results (from Seurat’s FindAllMarkers) were filtered for average log2 fold change (avg_log2FC) >= 0.25, percentage of expression (pct.1, pct.2) > 0.05 in each comparison group, and adjusted p-value (p_val_adj) <= 0.05. The “Local Network Cluster (STRING)” results from STRINGdb were used to compare patient v. control (in naïve T cells, memory T cells, and CSM B cells) and recurrent v. no recurrent infections in CSM B cells. For pathway enrichment in Tfh cells in recurrent v. no recurrent infections, the “Reactome Pathways” STRINGdb results were used. All STRINGdb pathway results were filtered by false discovery rate < 0.01 and plotted as dot plots using *ggplot2*^3^ (v3.5.1). In the STRINGdb dot plots, the plots are faceted based on keywords in the pathway names. In some cases, the pathway names have been truncated for legibility. Full pathway names and data values can be found in Supplemental Tables 2-6.

*Statistical analyses*

For Wilcoxon Rank Sum test of the differences in cell populations between different subject cohorts, the *wilcox.test* function from the base R package *stats*^2^ (v3.6.2) was used.

*Visualization of single cell data*

Single-cell UMAP plots were generated using the package *scplotter*^4^ (v0.10). The package *scCustomize*^5^ (v2.1.2) was used to generate the dot plots of gene expression (Figure 5B, Supplemental Figures 4 and 5). In Figure 5B, manual adjustments (colors of group annotations and dashed line to highlight Tfh cells) were made in Adobe Illustrator.

*Gene Ontology Enrichment*

Gene ontology (GO) enrichment of the “biological pathway” terms (as presented in Supplemental Figure 6) was performed using the *enrichGO* function in clusterProfiler^6^ (v4.16.0). GO enrichment analysis was performed using the differentially expressed genes for each indicated cell type that had an adjusted p-value < 0.05. In *enrichGO,* p-values were adjusted using the Benjamini-Hochberg procedure and p- and q-value cutoffs of 0.05.

*Senescence gene expression dot plots*

In the STRINGdb pathway analysis, the presence of senescence-related pathways was noted in the Reactome pathways results. Gene names for the following pathway terms were extracted from the STRINGdb Reactome results for Follicular helper T cells: R-HSA-2559582, 'Senescence-Associated Secretory Phenotype (SASP)'; R-HSA-2559580, 'Oxidative Stress Induced Senescence'; R-HSA-2559583, 'Cellular Senescence'. To visualize the expression of these genes across all cell types, the package *scCustomize*^5^ (v2.1.2) was used to visualize gene expression in a dot plot. Manual adjustments to aesthetics (colors of group and cell type annotations, legend placement) were made in Adobe Illustrator.

**References**

1. Szklarczyk D, Kirsch R, Koutrouli M, Nastou K, Mehryary F, Hachilif R, Gable AL, Fang T, Doncheva NT, Pyysalo S, Bork P, Jensen LJ, von Mering C. The STRING database in 2023: protein-protein association networks and functional enrichment analyses for any sequenced genome of interest. 2023. Nucleic Acids Res 6;51(D1):D638-D646. doi: 10.1093/nar/gkac1000. PMID: 36370105; PMCID: PMC9825434.
2. R Core Team (2024). R: A Language and Environment for Statistical Computing_. R Foundation for Statistical Computing, Vienna, Austria. <https://www.R-project.org/>.
3. Wickham H. 2016. ggplot2: Elegant Graphics for Data Analysis. Springer-Verlag New York.
4. Wang P. 2024. scplotter: Publication Quality Plots for Single Cell Data Analysis. R package version 0.1.0, commit 6de8852d77aaa4f0febde8afd49f7b31187ec74c, github.com/pwwang/scplotter.
5. Marsh SE. 2021. scCustomize: Custom Visualizations & Functions for Streamlined Analyses of Single Cell Sequencing. <https://doi.org/10.5281/zenodo.5706430>. RRID:SCR_024675.
6. Yu G, Wang LG, Han Y, He QY. clusterProfiler: an R package for comparing biological themes among gene clusters. OMICS. 2012 May;16(5):284-7. doi: 10.1089/omi.2011.0118. Epub 2012 Mar 28. PMID: 22455463; PMCID: PMC3339379.
